# Muscle hypertrophy and ladder‐based resistance training for rodents: A systematic review and meta‐analysis

**DOI:** 10.14814/phy2.14502

**Published:** 2020-09-05

**Authors:** Ítalo Lourenço, Walter Krause Neto, Laura dos Santos Portella Amorim, Vagner Moraes Munhoz Ortiz, Vitor Lopes Geraldo, Gabriel Henrique da Silva Ferreira, Érico Chagas Caperuto, Eliane Florencio Gama

**Affiliations:** ^1^ Department of Physical Education Laboratory of Morphoquantitative Studies and Immunohistochemistry Universidade São Judas Tadeu São Paulo SP Brazil; ^2^ Department of Physical Education Laboratory of Human Moviment Universidade São Judas Tadeu São Paulo SP Brazil

**Keywords:** cross‐sectional area, exercise, rats, skeletal muscle, strength training

## Abstract

This study aimed to review the effects of ladder‐based resistance training (LRT) on muscle hypertrophy and strength in rodents through a systematic review with meta‐analysis. We systematically searched PubMed/Medline, SportDiscuss, Scopus, Google Scholar, Science Direct, and Scielo database on May 18, 2020. Thirty‐four studies were included measuring total (mCSA) or mean muscle fibers cross‐sectional area (fCSA) or maximum load‐carrying capacity (MLCC) or muscle mass (MM). About the main results, LRT provides sufficient mechanical stimulation to increase mCSA and fCSA. Meta‐analysis showed a significant overall effect on the fCSA (SMD 1.89, 95% CI [1.18, 2.61], *p* < .00001, *I* ^2^ = 85%); however, subgroup analysis showed that some muscle types might not be hypertrophied through the LRT. Meta‐analysis showed a significant training effect on the MM (SMD 0.92, 95% CI [0.52, 1.32], *p* < .00001, *I* ^2^ = 72%). Sub‐group analysis revealed that soleus (SMD 1.32, 95% CI [0.11, 2.54], *p* = .03, *I* ^2^ = 86%) and FHL (SMD 1.92, 95% CI [1.00, 2.85], *p* < .0001, *I* ^2^ = 71%) presented significant training effects, despite moderate heterogeneity levels (*I* ^2^ = 72%). MLCC increases considerably after a period of LRT, regardless of its duration and the characteristics of the protocols (SMD 12.37, 95% CI [9.36, 15.37], *p* < .00001, *I* ^2^ = 90%). Through these results, we reach the following conclusions: (a) LRT is efficient to induce muscle hypertrophy, although this effect varies between different types of skeletal muscles, and; (b) the ability of rodents to carry load increases regardless of the type and duration of the protocol used.

## Abstract 2

Ladder resistance training (LRT) provides sufficient mechanical stimulation to induce increases in cross‐sectional area in most types of skeletal muscle; however, some types of muscles with certain morphological and biochemical characteristics may not be hypertrophied through the LRT. In general, the chronic response of LRT over skeletal muscle mass seems to vary between different types of muscles. The maximum load‐carrying capacity increases considerably after a period of LRT, regardless of its duration and the characteristics of the protocols.

## 1 INTRODUCTION

Primarily, resistance training is a modality that aims to improve strength and increase muscle mass (Ratamess et al., 2009; Schoenfeld, Contreras, Vigotsky, & Peterson, 2016). In humans, resistance training prescription control is conducted based on parameters such as volume [sets and repetitions] and intensity [load] (Ratamess et al., 2009). Also, exercise selection appears to be fundamental to the success of the training protocol, especially concerning its efficiency in stimulating muscle hypertrophy (Kubo, Ikebukuro, & Yata, 2019).

On the other hand, resistance training for rodents has always been a reason for debate (Cholewa et al., 2013; Krause Neto, Silva, Ciena, Anaruma, & Gama, 2016; Strickland & Smith, 2016). Such controversy was generated from the apparent differences found between the equipment used for the training of rodents and humans. However, since the ladder‐based resistance training model (LRT) proposed by Hornberger and Farrar (2004), a large amount of evidence has emerged, and greater control of training variables has been investigated and better controlled in experimental resistance training (Krause Neto et al., 2016, 2018; Luciano et al., 2017; Tibana et al., 2017). Despite this, there is still some doubt as to the efficiency of this training model in stimulating muscle hypertrophy of different muscle types in young and adult rats (Hornberger & Farrar, 2004; Son et al., 2016).

In 2004, Hornberger and Farrar investigated the effects of 8 weeks of LRT on the total muscle cross‐sectional area (mCSA), muscle mass, the total, and myofibrillar protein of the soleus, plantaris, flexor hallucis longus (FHL), gastrocnemius, and quadriceps femoris muscles of young rats. After collecting and analyzing the results, the authors demonstrated that only the FHL muscle showed a statistical change in these parameters. This fact raised a hypothesis about a probable inefficiency of this training model in stimulating muscle hypertrophy of more types of muscles, thus limiting the study on other outcomes. However, more recent evidence has shown that some of these outcomes, such as the measurement of muscle mass, might not be effective in showing the real effect of training and thus affect the interpretation of data and the efficiency of the training model. According to Tibana et al. (2017), despite no increased muscle mass, LRT is efficient in inducing a significant increase in the mean muscle fibers cross‐sectional area (fCSA) and the capacity of the rodent to carry the load. Yet, other studies corroborate these conclusions, demonstrating that LRT might stimulate hypertrophy of several different types of muscles (Jung et al., 2015; Know, Jang, Cho, Jang, & Lee, 2017; Lim, Gil, Quan, Viet, & Kim, 2018; Luciano et al., 2017; Ribeiro et al., 2017).

Due to the variability of results found in the literature, we find it convenient to carry out a more in‐depth investigation of the research associated with the LRT model. Therefore, the present study aimed to review the effects of LRT on muscle hypertrophy in rodents through a systematic review with meta‐analysis.

## 2 METHODS

This systematic review followed the PRISMA guideline [preferred reporting items for systematic reviews and meta‐analysis] (Liberati et al., 2009). This guideline is widely used in systematic reviews of clinical trials; however, it is possible to adapt this instrument to systematically review the literature covering animal studies (Hooijmans et al., 2014; Hooijmans, de Vries, Rovers, Gooszen, & Ritskes‐Hoiting, 2012; Krause Neto, Ciena, Anaruma, de Souza, & Gama, 2015). Therefore, on May 18th, 2020, we searched the PubMed/Medline, SportDiscuss, Scopus, Google Scholar, Science Direct and Scielo databases, using the following Mesh and entry terms and additional key words:((((resistance training OR training, resistance OR strength training OR training, strength OR weight‐lifting strengthening program OR strengthening program, weight‐lifting OR strengthening programs, weight‐lifting OR weight lifting strengthening program OR weight‐lifting strengthening programs OR weight lifting exercise program OR exercise program, weight‐lifting OR exercise programs, weight‐lifting OR weight lifting exercise program OR weight‐lifting exercise programs OR weight‐bearing strengthening program OR strengthening program, weight‐bearing OR strengthening programs, weight‐bearing OR weight bearing strengthening program OR weight‐bearing strengthening programs OR weight‐bearing exercise program OR exercise program, weight‐bearing OR exercise programs, weight‐bearing OR weight bearing exercise program OR weight‐bearing exercise programs OR resistance exercise))) AND (hypertrophy OR hypertrophies OR cell enlargement OR enlargement, cell OR cell size growth OR growth, cell size OR growths, cell size OR size growth, cell OR cell growth in size OR cross‐sectional area OR muscle strength OR force OR strength OR muscular strength OR torque OR muscular endurance OR lifting OR muscle contraction OR contraction, muscle OR contractions, muscle OR muscle contractions OR muscular contraction OR contraction, muscular OR contractions, muscular OR muscular contractions)) AND ((rats OR Rat OR rattus OR rattus norvegicus OR rats, norway OR rats, laboratory OR laboratory rat OR laboratory rats OR rat, laboratory OR mice OR Mus OR mouse OR mus musculus OR mice, house OR house mice OR mouse, house OR house mouse OR mus domesticus OR mus musculus domesticus OR domesticus, mus musculus OR mice, laboratory OR laboratory mice OR mouse, laboratory OR laboratory mouse OR mouse, swiss OR swiss mouse OR swiss mice OR mice, swiss OR wistar rats)).

### 2.1 Inclusion and exclusion criteria

For inclusion of articles, the following criteria were followed: (a) samples composed of rats aged 2–13 months; (b) resistance training protocol performed on the ladder‐based equipment (at least 1‐m height); (c) outcomes that included quantification of the mCSA OR fCSA OR the mass of the skeletal muscles OR the quantification of the maximum carried load measured by muscular endurance test OR the quantification of volume differences of load trained between the first and last training sessions, and (d) having a control group not submitted to the training model.

We excluded all articles that investigated exercise effects on mice, training without additional load, genetically modified animals, interventions such as surgery, muscle unloading or electrical shock stimulation, use of any drug or food supplement, different types of animal diet and studies with insufficient data or that used old animals (above 16 months of age at the beginning of the intervention). Rat lineage was not stated as an inclusion criterion.

### 2.2 Studies selection

The selection of studies was conducted by independent researchers (IL, WKN, LSPA, VMMO, VLG, and GHSF). After reading the titles and abstracts, a meeting determined the number of studies included for the full‐text analysis. A week later, investigators met again to identify the final number of studies included and to resolve any conflict of opinion about the selection process. Upon completing the ultimate selection of studies, an analysis of quality and risk of bias was initiated. When necessary, the corresponding author of the study was contacted to request further information.

### 2.3 Analysis of data quality, assessment of risk of bias, and publication bias

We assessed the risk of bias of the included studies using a questionnaire described elsewhere (Hooijmans et al., 2014; Hooijmans et al., 2012). We based these criteria on the possible presence of selection bias (questions 1, 2, and 3), performance bias (questions 4 and 7), detection bias (questions 5, 6, and 8), and attrition bias (questions 9 and 10). The quality analysis and risk of bias were independently assessed by two reviewers (IL and WKN), using predefined judging criteria (Hooijmans et al., 2012). The scores "Yes" indicate a low risk of bias; the score "No" indicates a high risk of bias, "Unclear" indicates an unknown risk of bias. To detect publication bias, funnel plots were created.

### 2.4 Data extraction

We extracted data about rodent lineage, age, gender, training parameters (MLCC and training protocols), primary outcomes, and main results.

### 2.5 Outcomes

The primary outcome was muscle hypertrophy [mCSA and fCSA]. The secondary outcomes were individual muscle mass (MM) and maximum load‐carrying capacity (MLCC).

### 2.6 Data synthesis and meta‐analysis

Systematic review data were organized in Tables 1 and 2. For the meta‐analysis, the mean and standard deviation values were extracted from each outcome. Studies that investigated the effects of LRT on more than one muscle type per result had a sequential number added to their identification in the forest plots (i.e., Padilha et al., 2019). The number of muscle samples analyzed in each study was added to the forest plots as a sample number. Meta‐analysis was applied for fCSA, MM, and MLCC. mCSA data was insufficient to run meta‐analysis. For statistical analysis, we used review manager software 5.3 to calculate the standardized mean difference ([SMD], the mean of the experimental group minus the mean of the control group divided by the pooled *SD* of the two groups), 95% confidence interval (95% CI) and heterogeneity by the *I* ^2^, Chi^2^, and Tau^2^ values. We used *I* ^2^ to assess heterogeneity between studies using random‐effect models (*I* ^2^ values <50% indicate low heterogeneity, 50%–75% moderate heterogeneity, and >75% high level of heterogeneity). Analysis of subgroups was applied as necessary. For the overall effect, *p* ≤ .05 was considered statistically significant.

**TABLE 1:** Description of studies included according to rat lineage, sample number per group, age, and initial body mass at the beginning of the experimental period, ladder equipment description, and outcomes of interest

Reference	Lineage	Sample Number per group	Age	Initial body mass	Ladder description	Muscles	Outcomes
Chi et al. (2020)	Sprague‐Dawley	10	8 weeks	Not described	1 m height and 85° inclination	Flexor hallucis longus and Flexor digitorum profundus	Muscle mass
							MLCT
							Mean fiber CSA
Padilha et al. (2019)	Wistar	7	Not described	210 ± 7.4 g	1.1 m height and 80° inclination	Plantaris, Soleus and Flexor hallucis longus	MLCT
							Mean fiber CSA
Perilhão et al. (2020)	Wistar	10	8–21 weeks	Not described	1.1 m height and 80° inclination	—	MLCT
Neves et al. (2019)	Wistar	5	12 weeks	378 ± 20 g (Control group)	1.1 m height and 80° inclination	Quadriceps femoris and Tibialis anterior	Muscle mass
				368 ± 26 g (Dynamic, trained group)			MLCT
				348 ± 40 g (Isometric trained group)			Mean fiber CSA
Lee et al. (2018)	Sprague‐Dawley	8	Not described	Not described	1 m height and 85° inclination	Flexor hallucis longus	Muscle mass
							Mean fiber CSA
Lim et al. (2018)	Sprague‐Dawley	7	10 weeks	Not described	1 m height and 85° inclination	Extensor digitorum longus	MLCT
							Mean fiber CSA
Kwon et al. (2018)	Wistar Hannover	10	14 weeks	Not described	1.15 m height and 85° inclination	Flexor digitorum profundus	Muscle mass
							MLCT
							Mean fiber CSA
Padilha et al. (2017)	Wistar	9	Not described	252.4 ± 19.4 g	1.10 m height and 90° inclination	Soleus	MLCT
							Mean fiber CSA
Souza et al. (2017)	Wistar	10	3 months	Not described	1.1 m height and 80° inclination	—	MLCT
Ribeiro et al. (2017)	Wistar	6	3 months	298.74 ± 32 g	1.1 m height and 80° inclination	Soleus and Gastrocnemius	Muscle mass
Tibana et al. (2017)	Wistar	4–5	5 months	384.5 ± 42.6 g (Control group)	1.1 m height and 80° inclination	Gastrocnemius	Muscle mass
				349.2 ± 32.2 g (4 sets group)			Mean fiber CSA
				368.8 ± 32.7 g (8 sets group)			
Luciano et al. (2017)	Wistar	6	3 months	Not described	1.1 m height and 80° inclination	Quadriceps femoris	Mean fiber CSA
Carbone et al. (2017)	Wistar	8	3 months	Not described	1.1 m height and 80° inclination	—	MLCT
Krause Neto and Gama (2017)	Wistar	5	13 months	526.0 ± 105.3 g	1.1 m height and 80° inclination	Soleus and Extensor digitorum longus	Mean fiber CSA
Gomes et al. (2017)	Wistar	8	8 weeks	Not described	Not described	—	MLCT
Antonio‐Santos et al. (2016)	Wistar	13–18	60 days	280.1 ± 9.3 g (Control group)	1.3 m height and 70° inclination	—	MLCT
				266.3 ± 10.1 g (Trained group)			
Lee et al. (2016)	Sprague‐Dawley	6	8 weeks	220 ± 5 g	1 m height and 85° inclination	Gastrocnemius, Soleus, Tibialis anterior and Flexor hallucis longus	Muscle mass
							Muscle CSA
Gil and Kim (2015)	Sprague‐Dawley	7	9 weeks	350 g	1 m height and 80° inclination	Flexor hallucis longus	Muscle mass
							MLCT
Mônico‐Neto et al. (2015)	Wistar	10	75 days	300−390 g	1.1 m height and 80° inclination	Plantaris	MLCT
							Muscle CSA
Jung et al. (2015)	Wistar	10	10 weeks	177.7 ± 4.4 (10 weeks old)	1 m height and 75° inclination	Tibialis anterior	Mean fiber CSA
			50 weeks	619.9 ± 21.4 (50 weeks old)			
Deschenes et al. (2015)	Fisher 344	10	9 months	352.7 ± 11.1 (Control group)	1 m height and 85° inclination	Soleus and Plantaris	Muscle mass
				315.4 ± 6.7 (Trained group)			Mean fiber CSA
Souza et al. (2014)	Wistar	8	13 weeks	250 ± 30 g	1.1 m height and 80° inclination	Biceps brachialis and gastrocnemius	Muscle mass
							MLCT
Grans et al. (2014)	Wistar	9	Not described	250–300 g	Not described	Soleus and Gastrocnemius	Muscle mass
							MLCT
Nascimento et al. (2013)	Wistar	5	13 months	526.0 ± 105.3 g	1.1 m height and 80° inclination	Triceps brachialis	Mean fiber CSA
Shamsi et al. (2013)	Wistar	8	Not described	250–280 g	1 m height and 80° inclination	Soleus and Flexor Hallucis Longus	Muscle mass
Cassilhas et al. (2013)	Wistar	10	90 days	300 g	1.1 m height and 80° inclination	Gastrocnemius, Flexor digitorum longus, Soleus and Plantaris	Mean fiber CSA
Deus et al. (2012)	Wistar	10	2–4 months	288 ± 22 g	1.1 m height and 80° inclination	—	MLCT
Prestes et al. (2012)	Wistar	10	3 months	250 ± 30 g	1.1 m height and 80° inclination	Soleus and Tibialis anterior	Mean fiber CSA
Domingos et al. (2012)	Sprague‐Dawley	6	Not described	220 ± 12 g	1.1 m height and 80° inclination	—	MLCT
Prestes et al. (2009)	Wistar	10	13 weeks	250 ± 30 g	1.1 m height and 80° inclination	—	MLCT
Hornberger and Farrar (2004)	Sprague‐Dawley	10	90 days	372 ± 10 g (Control group)	1.1 m height and 80° inclination	Flexor hallucis longus, Soleus, Plantaris, Gastrocnemius, quadríceps femoris	Muscle mass
				368 ± 9 g (Trained group)			MLCT
							Muscle CSA
Lee and Farrar (2003)	Sprague‐Dawley	5	5 months	Not described	1 m height and 85° inclination	Flexor hallucis longus, Soleus, Plantaris, Gastrocnemius	Muscle mass
							Muscle CSA
Deschenes et al. (2000)	Sprague‐Dawley	9	12 months	Not described	1 m height and 85° inclination	Soleus	Mean fiber CSA
Deschenes et al. (1994)	Sprague‐Dawley	8	Not described	~250 g	1 m height and 80° inclination	Soleus and Extensor digitorum longus	Muscle mass

Abbreviations: CSA, cross‐sectional area; MLCT, maximum load‐carrying test.

**TABLE 2:** Description of resistance training protocols and main findings

Reference	MLCT protocol	Training Duration	Training Protocol	Main findings (Statistical)
Chi et al. (2020)	‐50%, 75%, 90%, 100% bodyweight with subsequent 30 g increases until failure	10 weeks	‐10 climbs/session	↑Maximum carrying load capacity
	‐2 min interval between climbs		‐Initial load equals to 50% body weight plus 10% per session	↑Flexor Hallucis longus CSA
			‐3 days/week	
Padilha et al. (2019)	‐75% bodyweight with 30 g increases until failure	6 weeks	‐High‐load group (4–8 climbs): 50%, 75%, 90%, 100% bodyweight with subsequent 30 g increases until failure	↑MLCT (both groups)
	‐2 min interval between climbs		‐Moderate load‐group (8–16 climbs): 70% (weeks 1–2), 80% (weeks 3–4) and 85% (weeks 506) of the maximum carrying load.	↑Plantaris CSA (both groups)
			‐2 min intervals	↑Soleus CSA (both groups)
			‐3 days/week	
Perilhão et al. (2020)	‐50%, 75%, 90%, 100% bodyweight with subsequent 30 g increases until failure	12 weeks	‐3 training blocks compose of 4 weeks each (60%, 65%, 70%, and 75% of the maximum carrying load)	↑Maximum carrying load capacity
	‐2 min interval between climbs		‐12 climbs per session	
			‐90 s interval between climbs	
			‐5 days/week	
Neves et al. (2019)	‐75% bodyweight with 30 g increases until failure	12 weeks	‐8 sets of 1 min each with 30% of the maximum carrying load	↑Maximum carrying load capacity
	‐2 min interval between climbs		‐2 min interval between climbs	↑Quadriceps mean fibers CSA
			‐5 days/week	↑Tibialis anterior mean fibers CSA
Lee et al. (2018)	No	8 weeks	‐3 sets of 5 climbing repetitions (load equal to 50% to 300% body weight)	↑Flexor Hallucis Longus mean fibers CSA
			‐The 1‐min interval between repetitions and 2 min between sets	
			‐3 days/week	
Lim et al. (2018)	‐50%, 75%, 90%, 100% bodyweight with subsequent 30 g increases until failure	8 weeks	‐50%, 75%, 90%, 100% bodyweight with subsequent 30 g increases until failure	↑Maximum carrying load capacity
	‐2 min interval between climbs		‐2 min interval between climbs	↑Extensor Digitorum Longus mean fibers CSA
			‐3 days/week	
Kwon et al. (2017)	‐50% bodyweight with subsequent increases until failure	8 weeks	‐8 climbs (50% × 2 climbs, 75% × 2 climbs, 100% × 2 climbs and 100% + 30 g × 2 climbs)	↑Flexor Digitorum Profundus mean fibers CSA
	‐2 min interval between climbs		‐2 min interval between climbs	
			‐3 days/week	
Padilha et al. (2017)	‐75% bodyweight with 30 g increases until failure	25 sessions	‐50%, 75%, 90%, 100% bodyweight with subsequent 30 g increases until failure	↑Maximum carrying load capacity
	‐2 min interval between climbs		‐2 min interval between climbs	
			‐3 days/week	
Souza et al. (2017)	Not described	12 weeks	‐6 climbs (60% maximum carrying load)	↑Maximum carrying load capacity
			‐5 days a week	
Ribeiro et al. (2017)	‐75% bodyweight with 30 g increases until failure	12 weeks	‐65%, 85%, 90%, and 100% of each animal's maximum carrying capacity. If a rat reached 100% of its carrying capacity, an additional 30 g load would be added until failure	↑Gastrocnemius mean fibers CSA
	‐2 min interval between climbs		‐2 min interval between climbs	
			‐3 days/week	
Tibana et al. (2017)	‐75% bodyweight with 30 g increases until failure	8 weeks	‐Group 4 sets (50%, 75%, 90%, and 100% of each animal's maximum carrying capacity)	↑Gastrocnemius mean fibers CSA
	‐2 min interval between climbs		‐Group 8 sets (2 climbs on each intensity 50%, 75%, 90% and 100%);	‐More significant CSA increases were found in the 8 sets group. However, no differences (*p* = .970) were found between RT4 and RT8 groups when the CSA of the gastrocnemius muscle was normalized by the total body weight
			‐2 min interval between climbs;	
Luciano et al. (2017)	No	12 weeks	‐Endurance resistance training (ERT): a load of 10% of body weight, which was increased progressively to 20%, 30%, 40%, and 50% for 3–6 sets with 2‐min breaks and 12–15 repetitions	↑ Quadriceps mass in groups SRT and HRT
			‐Strength resistance training (SRT): load of 25% of body weight, which was increased progressively to 50%, 100%, 125%, 150%, 175%, and 200%, for 3–6 sets with a 2‐min break and 3–5 repetitions	↑Mean fibers CSA in all trained protocols, with a higher increase of CSA seen in response to an increase of exercise intensity
			‐Hypertrophy resistance training (HRT): a load of 25% of body weight, which was increased progressively to 50%, 75%, and 100%, for 3–6 sets with a 2‐min break and 8–10 repetitions	
Carbone et al. (2017)	‐75% bodyweight with 30 g increases until failure	8 weeks	‐GF1: 50% bodyweight	↑maximum load‐carrying capacity in both groups. Larger increases were seen for heavier loads
	‐2 min interval between climbs		‐GF2: 75% bodyweight	
			‐6 climbs	
			‐2 min interval between climbs	
			‐5 days/week	
Krause‐Neto and Gama (2017)	No	16 weeks	‐Load equal to 75% bodyweight during the 4 first weeks, increasing to 80%, 90%, 100%, 110% and 120% bodyweight until the end	↑Extensor digitorum longus and Soleus CSA
			‐6 climbs	
			‐5 days/week	
			‐45 s interval between climbs	
Gomes et al. (2017)	‐75% bodyweight with 50 g increases until failure	8 weeks	‐15 climbs/session	↑maximum load carrying capacity
	‐2 min interval between climbs		‐5 days/week	
			‐Load equals to 40%–60% maximal load	
			‐1 min interval between climbs	
Antonio‐Santos et al. (2016)	‐75% bodyweight with 30 g increases until failure	8 weeks	‐10 climbs/session	↑Maximum load‐carrying capacity
	‐2 min interval between climbs		‐5 days/week	
			‐Each session of training started with a load corresponding to 30% (first climb), 50% (second climb), and 80% (from third to 10th climb) of the individual maximum overload (measured at the week before)	
			‐90 s interval between climbs	
Lee et al. (2016)	No	36 weeks	‐Initial weight was 50% of the body weight; the load was increased by 10% of the bodyweight by each session. Stepping onto the ladder five times was one set, and the training was composed of five sets. The training regimen consisted of climbing ladder 5 × 3 sets, once every third day	↑Flexor hallucis longus CSA and mass
Gil and Kim (2015)	No	8 weeks	‐50%, 75%, 90%, 100% bodyweight with subsequent 30 g increases until failure	↑Maximum load‐carrying capacity
			‐2 min interval between climbs	↑Relative Flexor Hallucis Longus mass/body mass
			‐3 days/week	
Mônico‐Neto et al. (2015)	‐75% bodyweight with 30 g increases until failure	8 weeks	‐50%, 75%, 90%, 100% bodyweight with subsequent 30 g increases until failure	↑Maximum load‐carrying capacity
	‐2 min interval between climbs		‐The 1‐min interval between climbs	↑Plantaris mean fibers CSA and mass
			‐5 days/week	
Jung et al. (2015)	No	8 weeks	‐50%, 75%, 90%, and 100% maximal load from the previous exercise session. This procedure was repeated until eight climbs were achieved or until the rat failed to climb the entire length of the ladder	↑Tibialis anterior mass
			‐2 min interval	↑Quadriceps mean fibers CSA
Deschenes et al. (2015)	No	7 weeks	Each training session featured eight repetitions of ladder climbing, and added resistance was initially set at 50% of body mass with 30 g increments added weekly	↓Soleus wet mass
Souza et al. (2014)	‐75% bodyweight with 30 g increases until failure	12 weeks	‐50%, 75%, 90%, 100% bodyweight with subsequent 30 g increases until failure	↑maximum load‐carrying capacity
	‐2 min interval between climbs		‐2 min interval between climbs	
			‐3 days/week	
Grans et al. (2014)	Initial load of 75% of body weight with additions of 15% of body weight in subsequent climbs	3 months	‐5 days/week	↑maximum load carrying capacity
			‐15 climbs/session	
			‐1 min interval	
			‐40 to 60% of maximum load/climb	
Nascimento et al. (2013)	No	16 weeks	‐Load equal to 75% bodyweight during the 4 first weeks, increasing to 80%, 90%, 100%, 110%, and 120% bodyweight until the end	↑Triceps Brachilais mean fibers CSA
			‐6 climbs	
			‐5 days/week	
			‐45 s interval between climbs	
Shamsi et al. (2014)	No	5 weeks	Five sets of four repetitions, each with a 60‐s rest interval between the reps and 3 min between the sets per session. At 13 and 14 sessions, rats were decreased to three sets of five repetitions	↑FHL mass and mass‐to‐body mass ratio
Cassilhas et al. (2013)	No	8 weeks	‐Eight climbs (2 × 50%, 2 × 75%, 2 × 90% and 2 × 100% bodyweight)	↑gastrocnemius, flexor digitorum longus, and plantaris mean fibers CSA
			‐1 min interval	
			‐5 days/week	
Deus et al. (2012)	‐Rats climbed the first step with load‐free and subsequent climbs occurring 2 min after the previous climb, with the load progressively increasing by 10% BW (pretraining) and by 30% BW at the end (posttraining);	8 weeks	‐3 times/week	↑maximum load‐carrying capacity
	‐Loads of MRT were raised until the animal could no longer climb the ladder		‐Each training session consisted of 58 climbs requiring 8–12 dynamic movements per climb	
Prestes et al. (2012)	‐75% bodyweight with 30 g increases until failure	12 weeks	‐50%, 75%, 90%, 100% bodyweight with subsequent 30 g increases until failure	↑Tibialis anterior mean fibers CSA
	‐2 min interval between climbs		‐2 min interval between climbs	
			‐3 days/week	
Domingos et al. (2012)	‐75% bodyweight with 30 g increases until failure	10 weeks	‐65, 85, 95, and 100% of the rat's previous maximal carrying capacity During subsequent ladder climbs, an additional 30‐g was added until a new maximal carrying capacity was determined	↑maximum load‐carrying capacity
	‐2 min interval between climbs		‐3 days/week	
			‐2 min interval	
Prestes et al. (2009)	‐75% bodyweight with 30 g increases until failure	12 weeks	‐50%, 75%, 90%, 100% bodyweight with subsequent 30 g increases until failure	↑maximum load carrying capacity
	‐2 min interval between climbs		‐2 min interval	
			‐3 days/week	
Hornberger and Farrar (2004)	‐75% bodyweight with 30 g increases until failure	8 weeks	‐50%, 75%, 90%, 100% bodyweight with subsequent 30 g increases until failure	↑Load carrying capacity
	‐2 min interval between climbs		‐2 min interval between climbs	↑Flexor Hallucis Longus mass
			‐3 days/week	
Lee and Farrar (2003)	No	8 weeks	‐Initial load of 50% body weight	↑Load carrying capacity
			‐3 sets of 5 reps	↑Flexor Hallucis Longus mass
			‐Intervals of 1 min between reps and 2 min between sets	
Deschenes et al. (2000)	No	7 weeks	‐10 climbs	No changes
			‐2 min rest intervals	
			‐3 days/week;	
			‐The weight attached to the tail sleeve was gradually increased from 50 g during the first session, to 535 g after the training program	
Deschenes et al. (1994)	No	11 weeks	‐8 climbs	↑Soleus wet mass and mass‐to‐body mass ratio
			‐2 min intervals	
			‐The resistance applied to the rats was progressively increased by 50 g every other week so that at the end of the program the resistance carried by the animals was 250 g, in addition to body weight	
			‐3 days/week	

Abbreviations: LCT, maximum load‐carrying test.

## 3 RESULTS

### 3.1 General data

#### 3.1.1 Description of the included studies

**FIGURE 1:**
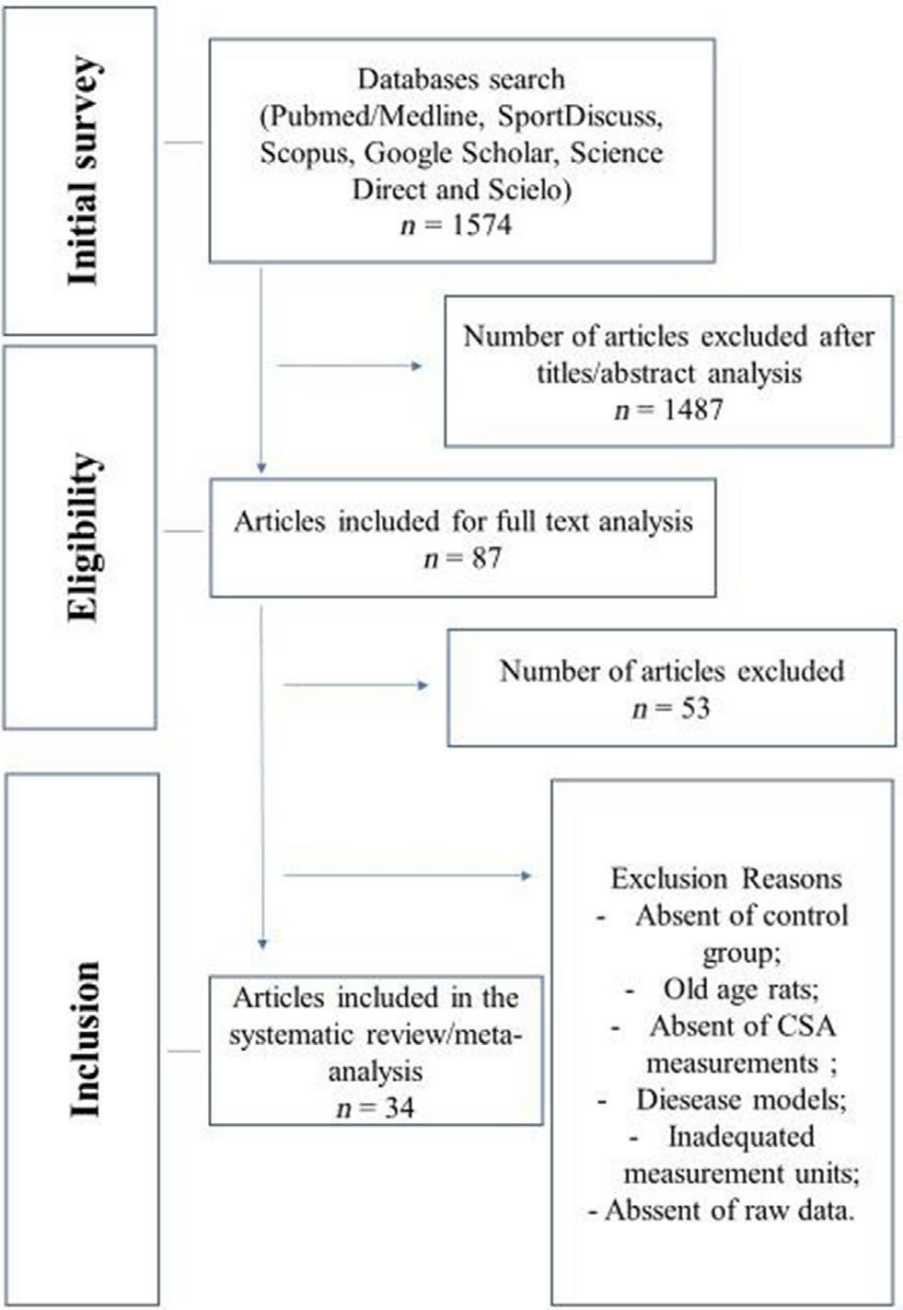
Flow gram of the articles selection process

After the initial search, we identified 1,574 articles titles. From this point, independent evaluators read all titles and abstracts, selecting 87 articles for full‐text analysis. After inclusion and exclusion criteria, 34 papers were included for systematic review (Figure 1).

Tables 1 and 2 present the overall data and main results of each selected article. Sprague–Dawley, Wistar, and Fisher 344 rats were the rodent lineages used within the studies. A total of 544 rats (434 male and 110 female) were included summing all studies. Rodent sample number per group varied from 4 to 18 rats. Twenty‐three articles described the rat's initial body mass (Antonio‐Santos et al., 2016; Cassilhas et al., 2013; Deus et al., 2012; Deschenes et al., 1994; Deschenes, Sherman, Roby, Glass, & Harris, 2015; Domingos et al., 2012; Gil & Kim, 2015; Grans et al., 2014; Hornberger & Farrar, 2004; Jung et al., 2015; Krause Neto & Gama, 2017; Lee, Hong, & Kim, 2016; Mônico‐Neto et al., 2015; Nascimento et al., 2013; Neves et al., 2019; Padilha et al., 2017; Padilha et al., 2019; Prestes et al., 2009, 2012; Ribeiro et al., 2017; Shamsi et al., 2013; Souza et al., 2014; Tibana et al., 2017). Ladder‐based equipment structure varied from 1–1.3 m of height and 70–90° of inclination between studies.

The muscles included in the selected articles were quadriceps femoris (Luciano et al., 2017; Neves et al., 2019), soleus (Cassilhas et al., 2013; Deschenes et al., 1994, 2000, 2015; Grans et al., 2014; Krause Neto & Gama, 2017; Padilha et al., 2017; Padilha et al., 2019; Prestes et al., 2012; Ribeiro et al., 2017; Shamsi et al., 2013), tibialis anterior [TA] (Jung et al., 2015; Lee et al., 2016; Neves et al., 2019; Prestes et al., 2012), gastrocnemius (Cassilhas et al., 2013; Grans et al., 2014; Lee et al., 2016; Ribeiro et al., 2017; Souza et al., 2014; Tibana et al., 2017), plantaris (Cassilhas et al., 2013; Deschenes et al., 2015; Mônico‐Neto et al., 2015; Padilha et al., 2019), FHL (Chi, Hou, Wu, Wang, & Yu, 2020; Gil & Kim, 2015; Hornberger & Farrar, 2004; Lee et al., 2016, 2018; Lee & Farrar, 2003; Padilha et al., 2019; Shamsi et al., 2014), extensor digitorum longus [EDL] (Deschenes et al., 1994; Krause Neto & Gama, 2017; Lim et al., 2018), flexor digitorum profundus [FDP] (Chi et al., 2020; Kwon et al., 2018), flexor digitorum longus [FDL] (Cassilhas et al., 2013), tríceps brachialis (Nascimento et al., 2013) and bíceps brachialis (Souza et al., 2014).

#### 3.1.2 Quality of reporting, risk of bias, and publication bias

**FIGURE 2:**
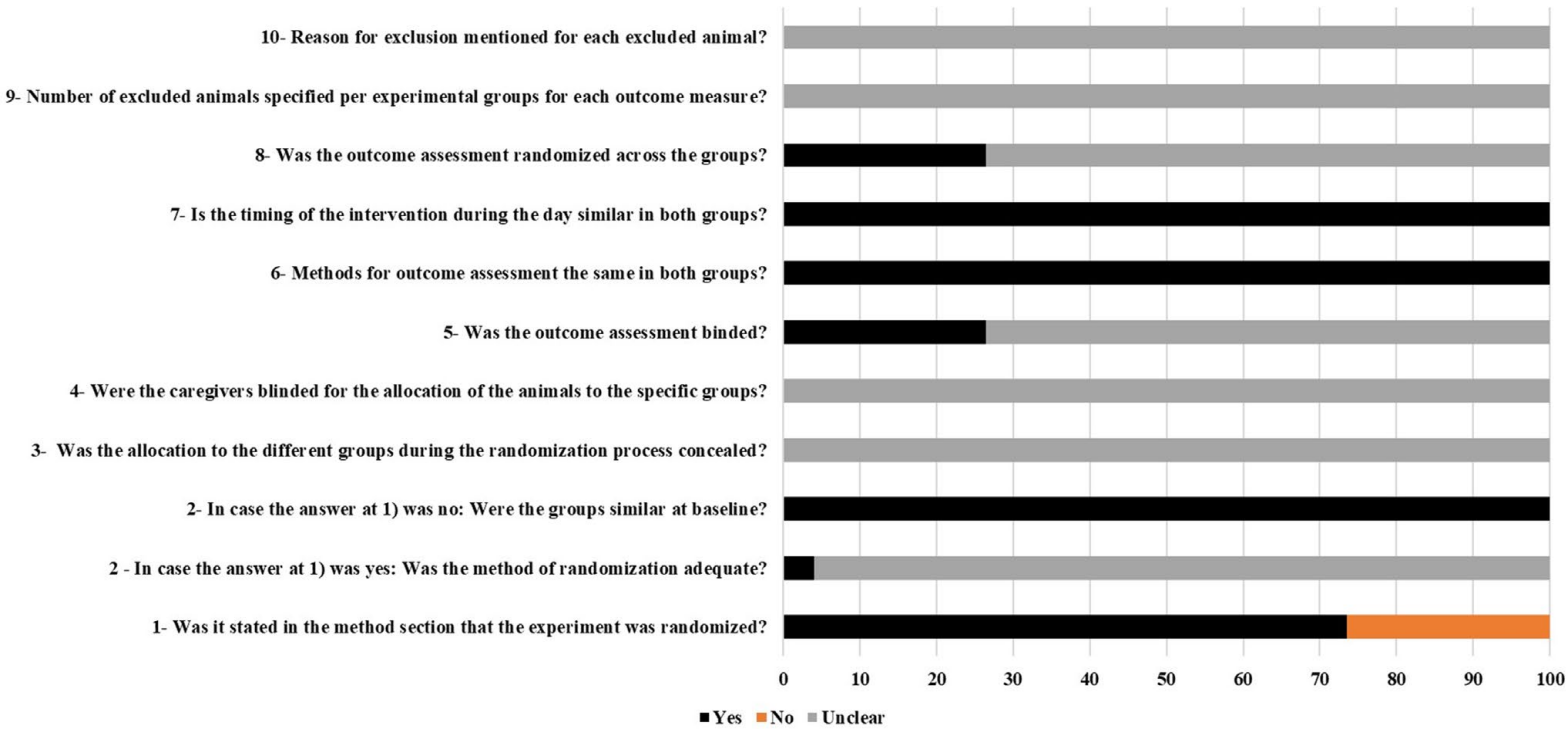
Risk of bias averaged per question. Legend: Yes = low risk of bias; No = high risk of bias; Unclear = unclear risk of bias

Figure 2 shows the average results of the risk of bias assessment. In all, 73.53% of the studies stated that the allocation of experimental units to treatment groups was randomized. However, only one study mentioned the randomization method (Padilha et al., 2019); nevertheless, all studies presented the division of groups in a similar way. None of the included articles described whether the allocation of groups during the randomization process was hidden or whether the caregivers knew which groups the animals were from. Only five studies reported having blinded the evaluation of results (Cassilhas et al., 2013; Deschenes et al., 2000, 2015; Luciano et al., 2017; Tibana et al., 2017). Also, 73.5% of studies did not describe whether there was randomization in the investigation of outcomes between groups. No study reported whether there was any sample loss throughout the training intervention. The quality scores varied between 4 and 6 points ("Yes" score). Only four studies scored 6 points (Cassilhas et al., 2013; Deschenes et al., 2015; Luciano et al., 2017; Tibana et al., 2017).

**FIGURE 3:**
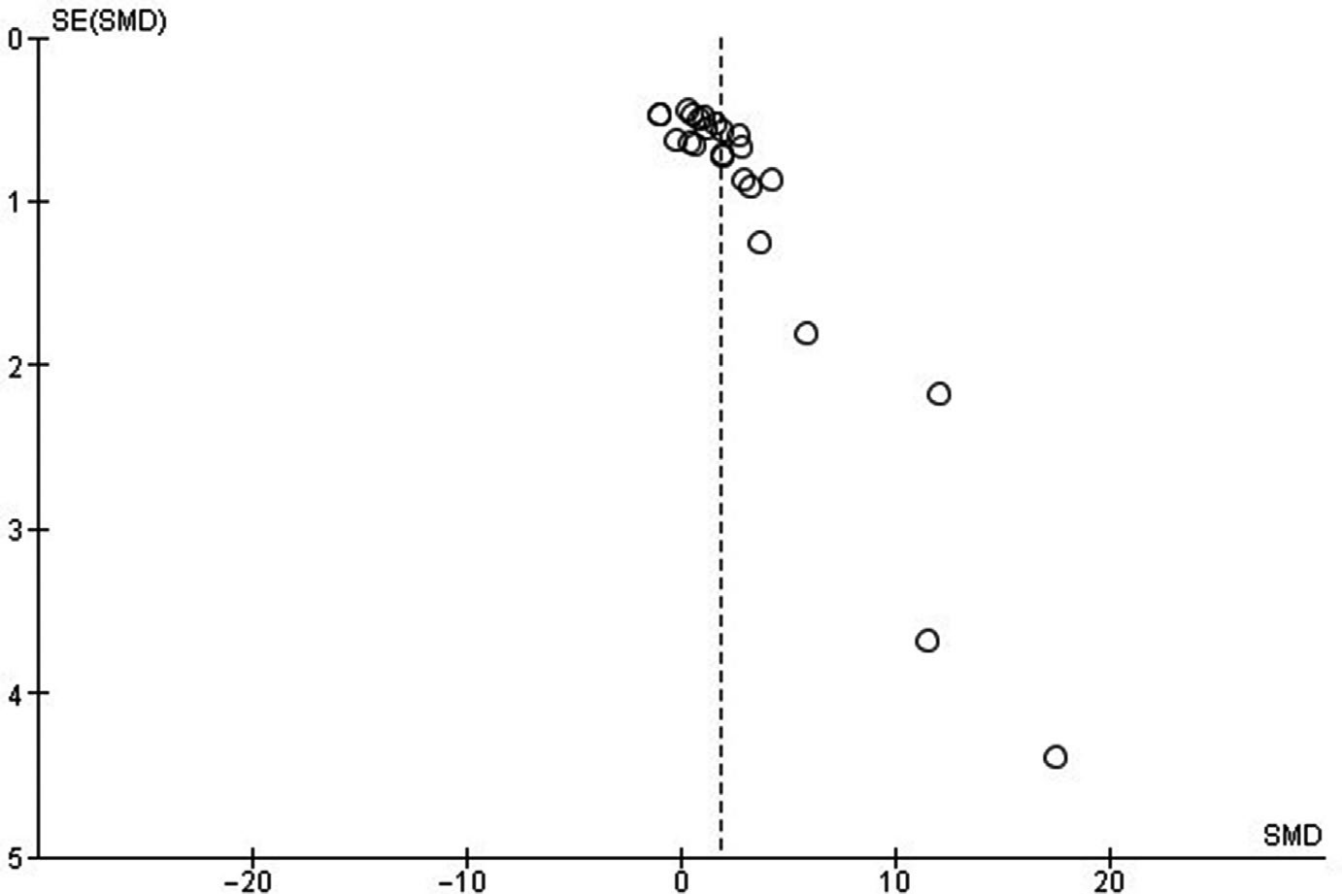
Funnel plot of standardized mean differences (SMD) of muscle fiber cross‐sectional area (fCSA). *SE* = standard error

**FIGURE 4:**
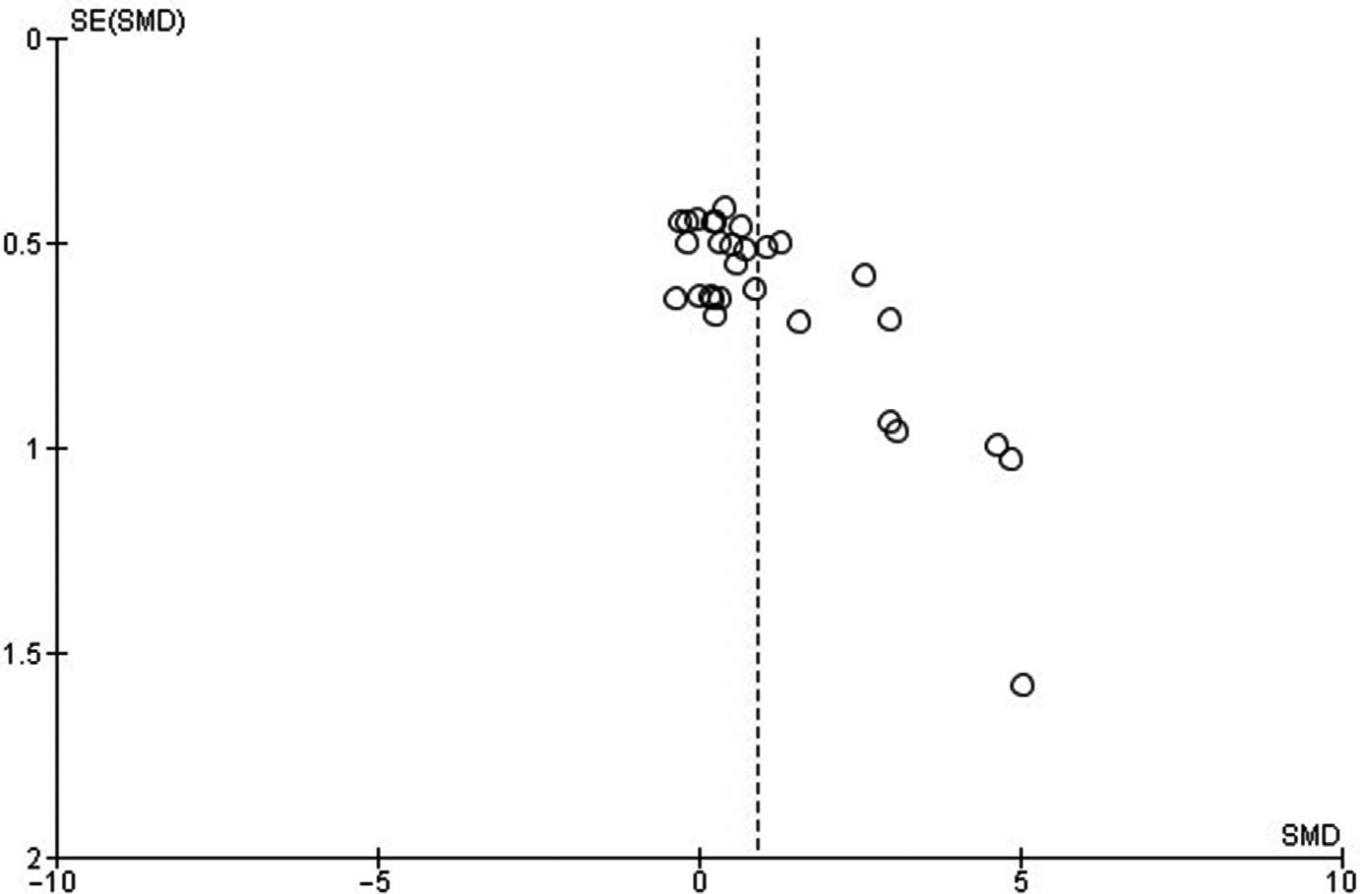
Funnel plot of standardized mean differences (SMD) of muscle mass (MM). *SE* = standard error

**FIGURE 5:**
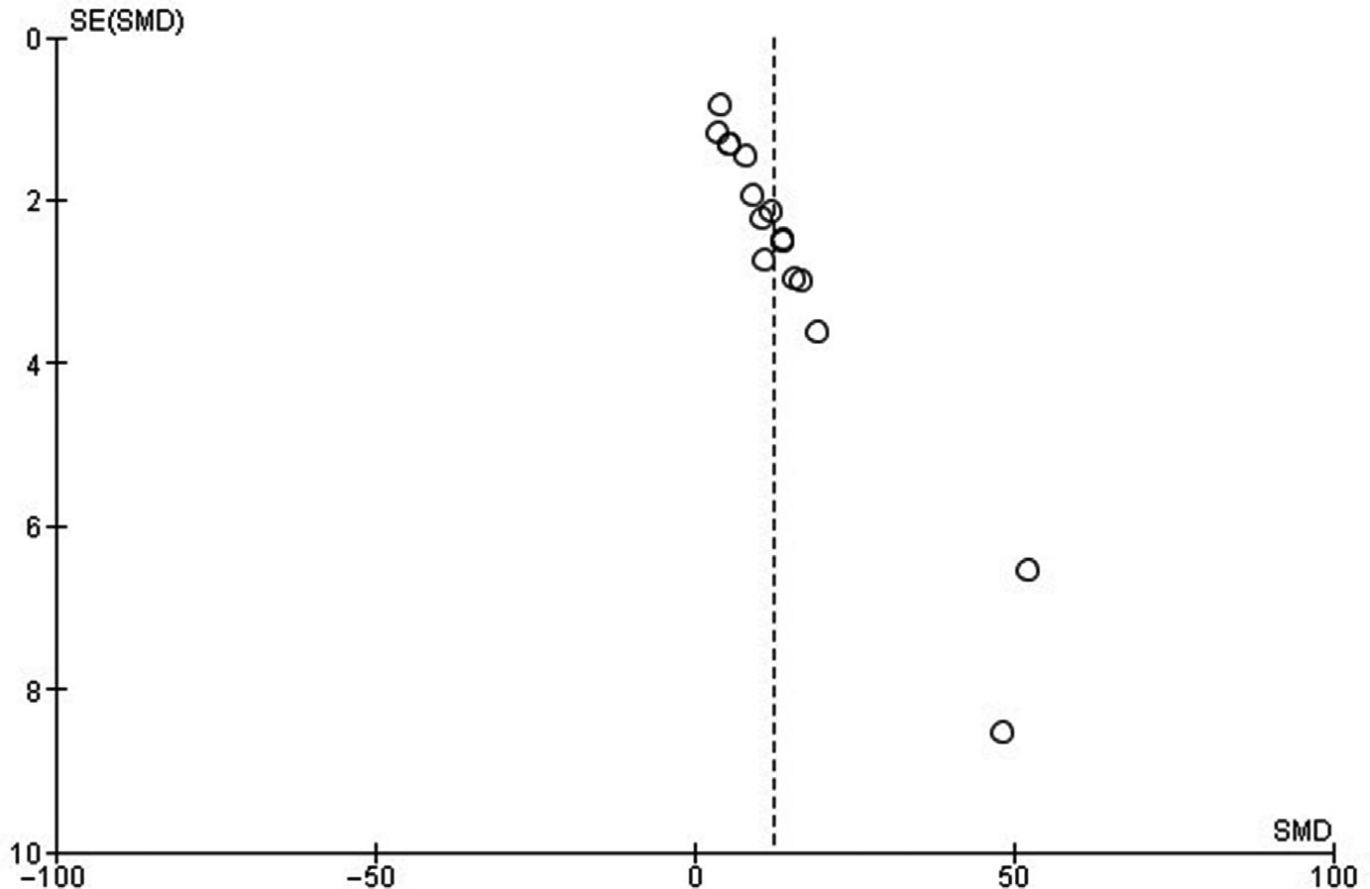
Funnel plot of standardized mean differences (SMD) of maximum load‐carrying capacity (MLCC). *SE* = standard error

The presence of publication bias was assessed for the outcomes fCSA, MM, and MLCC. Funnel plots demonstrated asymmetries for all three issues analyzed (Figures 3, 4, 5).

#### 3.1.3 Mean cross‐sectional muscle area

Mean mCSA was quantified by four articles (Hornberger & Farrar, 2004; Lee & Farrar, 2003; Lee et al., 2016; Mônico‐Neto et al., 2015). Of these, two studies reported a significant increase in the FHL muscle mCSA after LRT (Hornberger & Farrar, 2004; Lee & Farrar, 2003), while another article cited no difference (Lee et al., 2016). In addition to these, Mônico‐Neto et al. (2015) demonstrated an increase in mCSA of the plantaris muscle after LRT.

#### 3.1.4 Mean muscle fiber cross‐sectional area

Considering the analysis of myofibers hypertrophy, 16 articles measured the mean fCSA (Cassilhas et al., 2013; Chi et al., 2020; Deschenes et al., 2000, 2015; Jung et al., 2015; Krause Neto & Gama, 2017; Kwon et al., 2018; Lee et al., 2018; Lim et al., 2018; Luciano et al., 2017; Nascimento et al., 2013; Neves et al., 2019; Padilha et al., 2017; Padilha et al., 2019; Prestes et al., 2012; Tibana et al., 2017).

About soleus, two articles demonstrated significantly larger fCSA (Krause Neto & Gama, 2017; Padilha et al., 2019), while others did not show any change (Cassilhas et al., 2013; Deschenes et al., 2000, 2015; Padilha et al., 2017; Prestes et al., 2012).

About quadriceps femoris, two articles demonstrated fCSA hypertrophy (Luciano et al., 2017; Neves et al., 2019).

About TA, three articles showed fCSA hypertrophy (Jung et al., 2015; Neves et al., 2019; Prestes et al., 2012).

About gastrocnemius, two articles showed fCSA hypertrophy (Cassilhas et al., 2013; Tibana et al., 2017).

About EDL, one article showed fCSA hypertrophy (Lim et al., 2018), while others did not (Krause Neto & Gama, 2017).

About plantaris, two articles showed fCSA hypertrophy (Cassilhas et al., 2013; Padilha et al., 2019), while others did not (Deschenes et al., 2015).

About FHL, three articles showed fCSA hypertrophy (Chi et al., 2020; Lee et al., 2018; Padilha et al., 2019).

About FDL, one article showed fCSA hypertrophy (Cassilhas et al., 2013).

About FDP, one article showed fCSA hypertrophy (Know et al., 2017).

About tríceps brachialis, one article showed fCSA hypertrophy (Nascimento et al., 2013).

**FIGURE 6:**
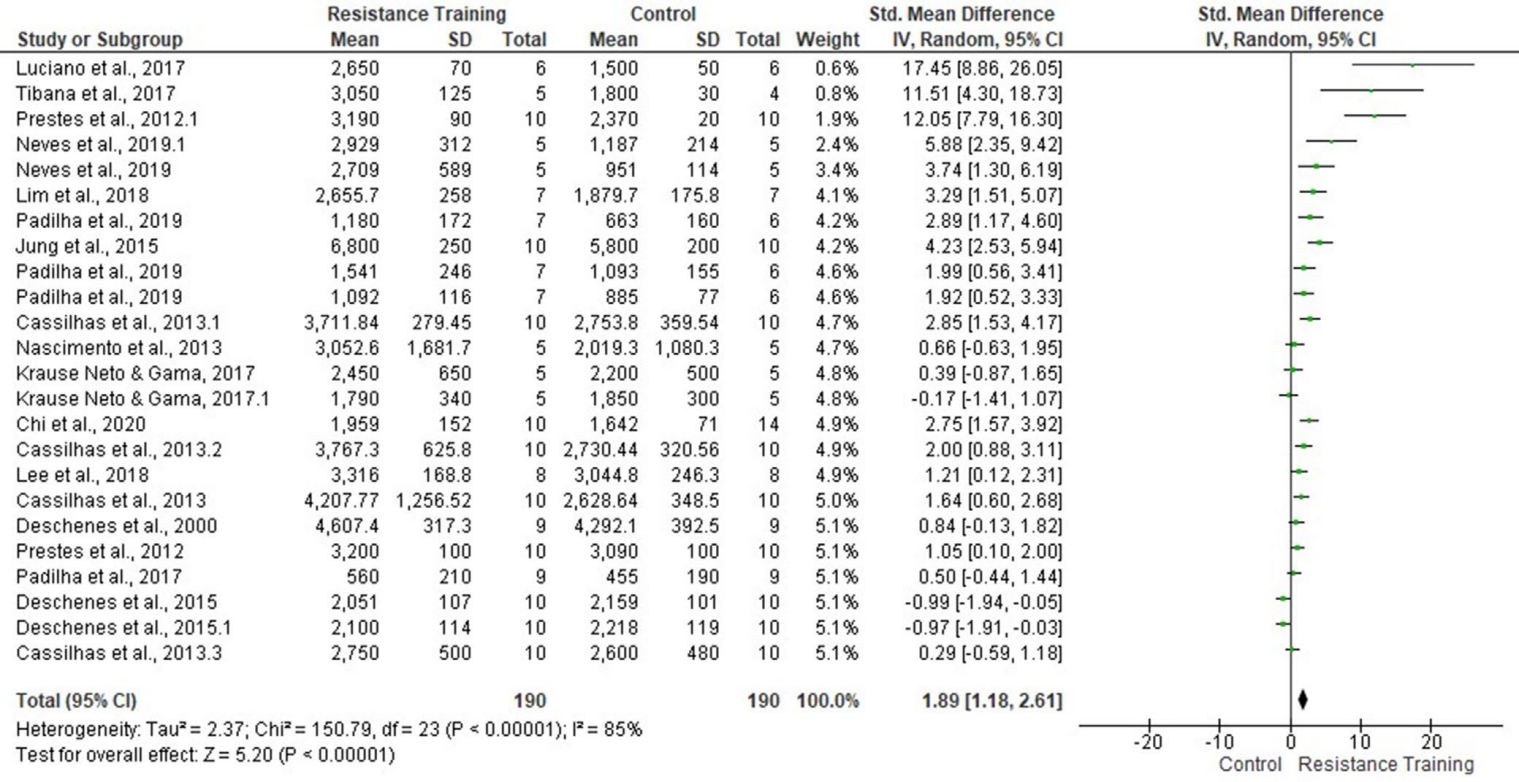
Forest plots of the data examining the effect of ladder resistance training on mean muscle fiber cross‐sectional area [fCSA] (produced in the review manager 5.3 software)

Muscle fibers hypertrophy was confirmed by meta‐analysis (15 studies, Figure 6). LRT groups demonstrated larger muscle fCSA (SMD 1.89, 95% CI [1.18, 2.61], *p* < .00001). However, a high heterogeneity level was found between studies (*p* < .00001, *I* ^2^ = 85%).

Due to the high degree of heterogeneity, subgroup analyses on individual skeletal muscles fCSA and training duration were applied.

**FIGURE 7:**
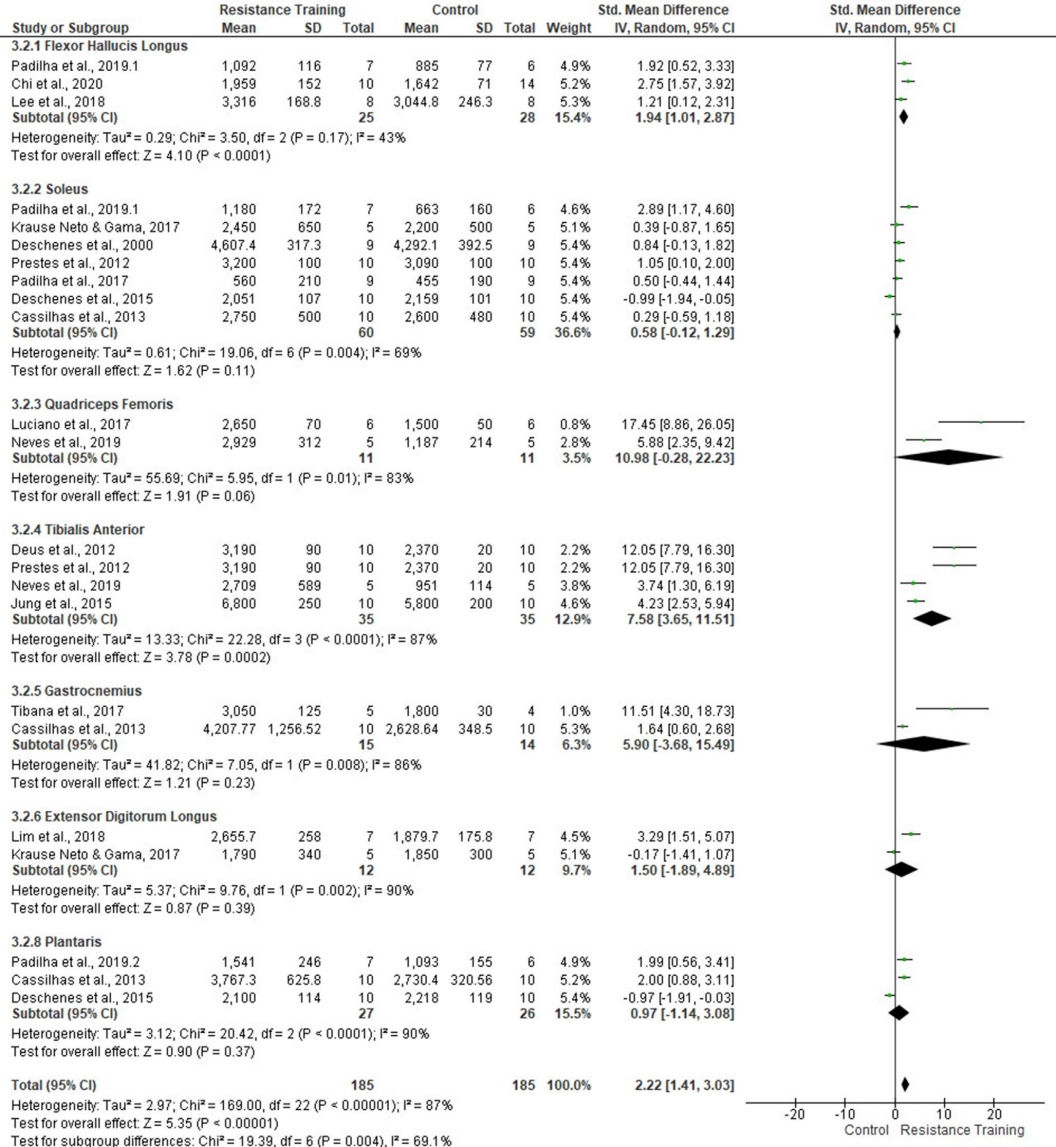
Forest plots of the data examining the effect of ladder resistance training on mean muscle fiber cross‐sectional area [fCSA] per muscle type (produced in the review manager 5.3 software)

In Figure 7, sub‐group analyses on the individual muscles fCSA revealed different training responses between muscle types. FHL (three studies, SMD 1.94, 95% CI [1.01, 2.87], *p* < .0001, *I* ^2^ = 43%) and TA (4 studies, SMD 7.58, 95% CI [3.65, 11.51], *p* = .0002, *I* ^2^ = 87%) muscles presented a statistical training effect on fCSA. However, quadriceps femoris (two studies, *p* = .06), soleus (four studies, *p* = .11), gastrocnemius (two studies, *p* = .23), EDL (two studies, *p* = .39), and plantaris (three studies, *p* = .47) muscles did not present significant overall effect. Despite these circumstances, the studies still showed moderate‐high heterogeneity. Clearly, the number of tissue samples taken from each muscle also appears to interfere with the results of this meta‐analysis (ie. quadríceps femoris and gastrocnemius).

**FIGURE 8:**
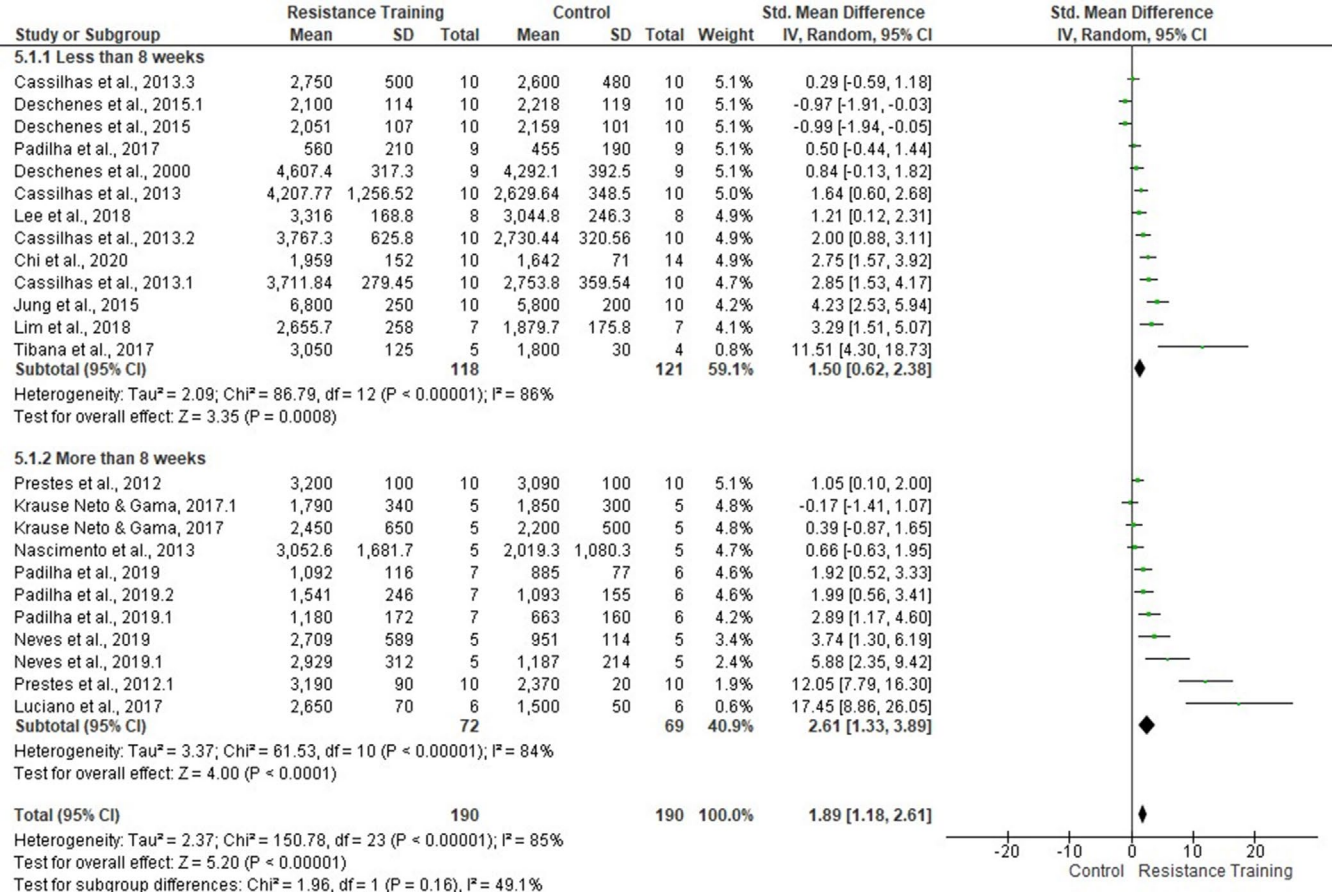
Forest plots of data examining the effect of ladder resistance training duration on mean muscle fiber cross‐sectional area [fCSA] (produced in the review manager 5.3 software)

About the influence of training duration on the muscle fCSA, nine articles presented evidence on muscle fCSA values for training periods until 8 weeks (SMD 1.5, 95% CI [0.62, 2.38], *p* = .0008, *I* ^2^ = 86%) and six articles above this period (SMD 2.61, 95% CI [1.33, 3.89], *p* < .0001, *I* ^2^ = 84%). Larger effects were found for periods over 8 weeks of training; however, this analysis also showed a high heterogeneity level (*I* ^2^ = 85%) [Figure 8].

#### 3.1.5 Muscle mass

Considering the muscle mass, 16 articles measured individual skeletal MM (Chi et al., 2020; Deschenes et al., 1994, 2015; Gil & Kim, 2015; Grans et al., 2014; Hornberger & Farrar, 2004; Know et al., 2018; Lee et al., 2016, 2018; Lee & Farrar, 2003; Neves et al., 2019; Ribeiro et al., 2017; Shamsi et al., 2014; Souza et al., 2014; Tibana et al., 2017).

About soleus, three articles showed MM increase (Deschenes et al., 1994; Grans et al., 2014; Lee et al., 2016), while five articles did not (Deschenes et al., 2015; Hornberger & Farrar, 2004; Lee & Farrar, 2003; Ribeiro et al., 2017; Shamsi et al., 2014).

About gastrocnemius, one article showed MM increase (Grans et al., 2014), while six articles failed (Hornberger & Farrar, 2004; Lee & Farrar, 2003; Lee et al., 2016; Ribeiro et al., 2017; Souza et al., 2014; Tibana et al., 2017).

About FHL, five articles showed greater MM (Hornberger & Farrar, 2004; Lee & Farrar, 2003; Lee et al., 2016; Lim et al., 2018; Shamsi et al., 2013), while other failed (Chi et al., 2020; Gil & Kim, 2015).

About TA, two articles showed no change in MM (Lee et al., 2016; Neves et al., 2019).

About plantaris, three articles showed that MM does not change (Deschenes et al., 2015; Hornberger & Farrar, 2004; Lee & Farrar, 2003).

About quadríceps, two articles failed to change MM (Hornberger & Farrar, 2004; Neves et al., 2019).

About EDl, one article showed no change (Deschenes et al., 1994).

About FDP, two articles showed no change (Chi et al., 2020; Kwon et al., 2018).

About bíceps brachialis, one article showed no change (Souza et al., 2014).

**FIGURE 9:**
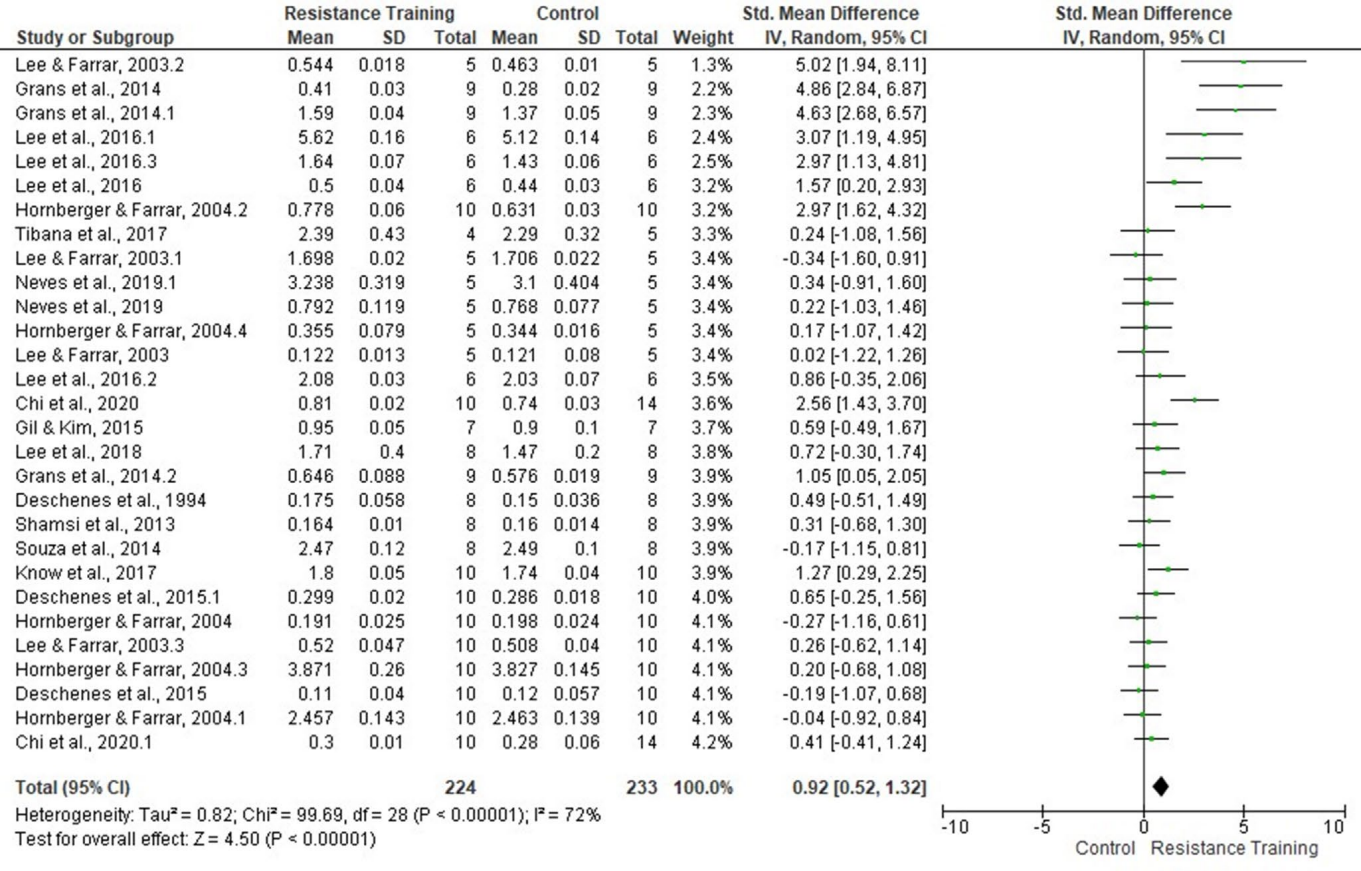
Forest plots of data examining the effect of ladder resistance training on muscle mass [MM] (produced in the review manager 5.3 software)

Meta‐analysis showed a significant training effect on the MM (SMD 0.92, 95% CI [0.52, 1.32], *p* < .00001). The forest plot (Figure 9) shows that the studies presented a moderated heterogeneity level (*p* < .00001, *I* ^2^ = 72%).

**FIGURE 10:**
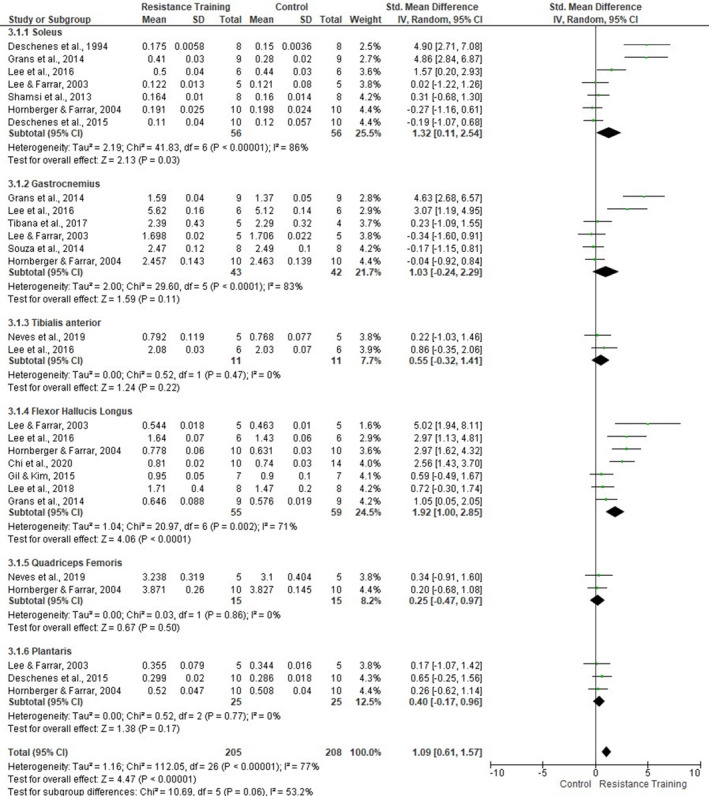
Forest plots of data examining the effect of ladder resistance training on individual muscle mass [MM] (produced in the review manager 5.3 software)

Sensitivity analysis was applied to each skeletal muscle (Figure 10). Subgroup analysis revealed that soleus (seven studies, SMD 1.32, 95% CI [0.11, 2.54], *p* = .03, *I* ^2^ = 86%) and FHL (seven studies, SMD 1.92, 95% CI [1.00, 2.85], *p* < .0001, *I* ^2^ = 71%) presented significant overall effect. However, no other muscle had a significant overall effect. The degree of heterogeneity for the included studies that assessed the MM of the TA and quadriceps femoris was considered low (*I* ^2^ = 0%). For all others, the degree of heterogeneity remained moderate‐high.

#### 3.1.6 Maximum load carrying capacity

The MLCC protocol most described within the studies used an initial load equals to 75% of the rodent bodyweight with 30 g increases until failure (2 min interval between climbs). However, considerable variability of training protocols was found here. The most common training protocol was described by Hornberger and Farrar (2004) [progressive loading increases from 50%, 75%, 90%, and 100% body weight with subsequent 30 g increases until failure; 2 min interval; and training frequency of 3 days/week]. Training duration varied from 6 to 36 weeks between studies.

**FIGURE 11:**
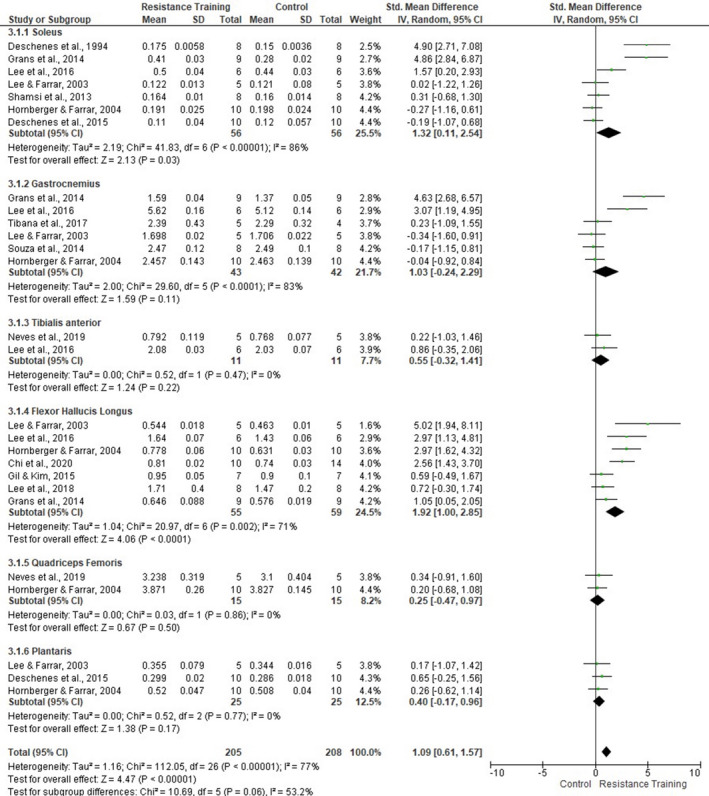
Forest plots of pre‐post training data examining the effect of ladder resistance training on maximum load‐carrying capacity [MLCC] (produced in the review manager 5.3 software)

**FIGURE 12:**
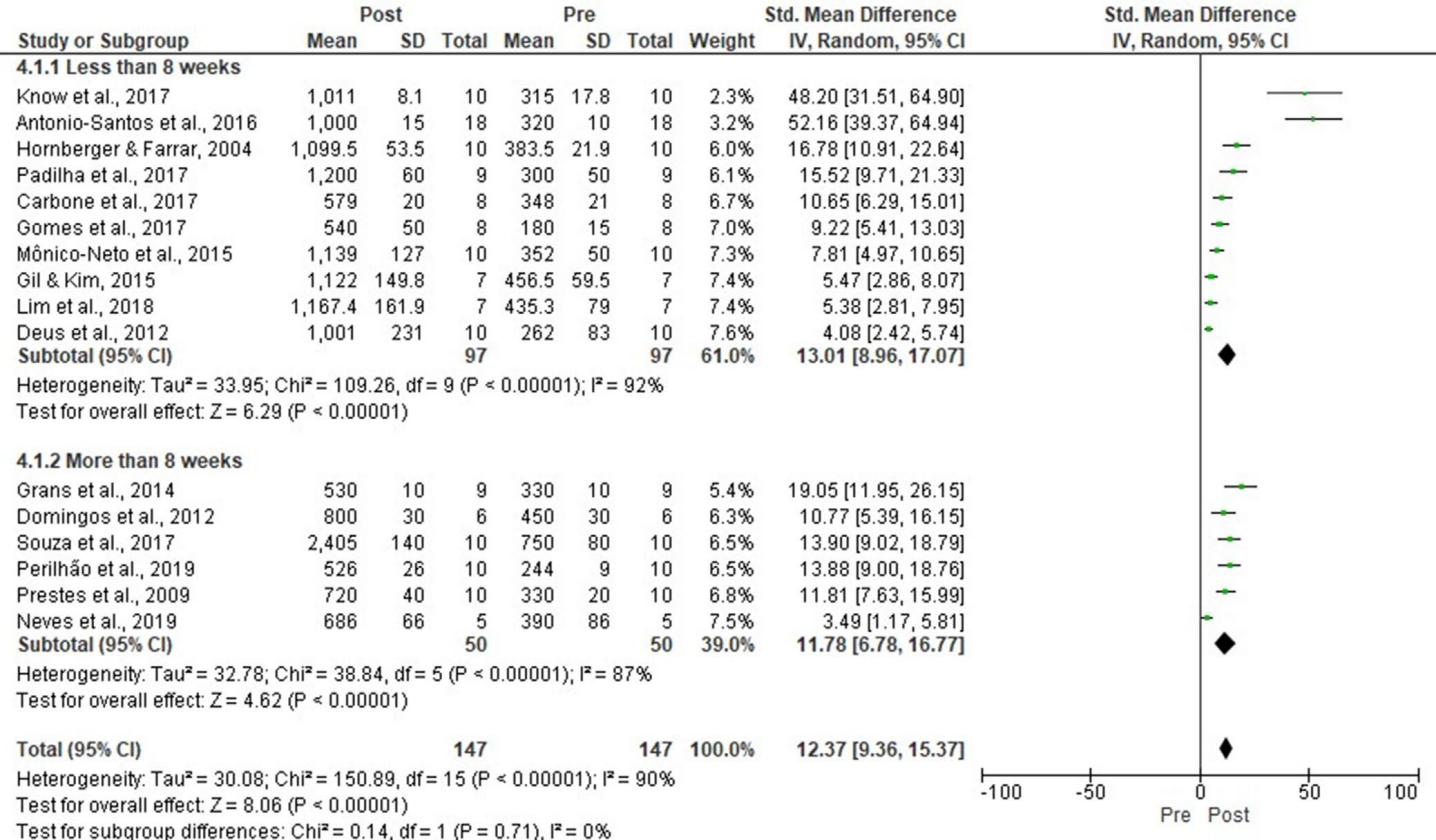
Forest plots of pre‐post training data examining the effect of ladder resistance training duration on maximum load‐carrying capacity [MLCC] (produced in the review manager 5.3 software)

Nineteen articles published data about the maximum load‐carrying tests protocol (Antonio‐Santos et al., 2016; Carbone et al., 2017; Chi et al., 2020; Deus et al., 2012; Domingos et al., 2012; Gil & Kim, 2015; Gomes, Borges, Rossi, Moura, & Medeiros, 2017; Grans et al., 2014; Hornberger & Farrar, 2004; Kwon et al., 2018; Lim et al., 2018; Mônico‐Neto et al., 2015; Neves et al., 2019; Padilha et al., 2017; Padilha et al., 2019; Perrilhão et al., 2019; Prestes et al., 2009; Souza et al., 2014; Souza et al., 2017). LRT presented a positive effect on the maximum load‐carrying capacity (16 studies, SMD 12.37, 95% CI [9.36, 15.37], *p* < .00001, *I* ^2^ = 90%, Figure 11). Sensitivity analysis was applied to LRT duration (Figure 12). Ten articles trained the rats until 8 weeks (SMD 13.01, 95% CI [8.96, 17.07], *p* < .00001, *I* ^2^ = 92%) and six articles above this period (SMD 11.78, 95% CI [6.78, 16.77], *p* < .00001, *I* ^2^ = 87%). Both training duration presented similar overall effects and high heterogeneity levels.

## 4 DISCUSSION

This systematic review demonstrated the following main results: (a) the methodological quality of the studies that investigated the effects of LRT on muscle hypertrophy in rodents needs to be improved; (b) LRT provides sufficient mechanical stimulation to induce increases in mCSA and fCSA in most types of skeletal muscle; however, some muscle types with specific morphological and biochemical characteristics may not be hypertrophied through the LRT (e.g., soleus); (c) in general, the chronic response of LRT over skeletal MM seems to vary between different types of muscles; and (d) MLCC increases considerably after a period of LRT, regardless of its duration and the characteristics of the protocols.

The quality analysis of the included studies was considered moderate in this systematic review. Most studies reported that groups were randomized during interventions; however, only one study cited how this process was conducted (Padilha et al., 2019). We can assume that in many laboratories, it is common to randomize the groups only after the MLCC tests. This attitude aims to subject to training only animals that were able to climb the ladder. In this way, randomization is done for convenience. However, the lack of adequate information on this criterion may raise questions about the sample selection bias. Therefore, we suggest that the authors fully describe how to sample randomization processes are being conducted.

The lack of description regarding blinding analysis of muscle tissue samples also raises some degree of concern. Only five studies reported that the histological slides were coded for blind analysis of the results (Cassilhas et al., 2013; Deschenes et al., 2000, 2015; Luciano et al., 2017; Tibana et al., 2017). The absence of blinding of the samples demonstrates a critical bias. Also, funnel plots showed moderate publication bias through asymmetries in the three outcomes investigated in this study. Specifically, in the fCSA result, three studies influenced this issue (Luciano et al., 2017; Prestes et al., 2012; Tibana et al., 2017). The use of a few rodents by groups and small measures of dispersion may have influenced this question.

The increase in MM and CSA, as a chronic response to resistance training, is not a new outcome in the literature. However, many questions were asked about the efficiency and effectiveness of training equipment and protocols, used in research with rodents, to stimulate significant changes in the structure and composition of skeletal muscles (Cholewa et al., 2013; Krause Neto et al., 2016). Here, we demonstrated that LRT is efficient to induce significant increases in mCSA and fCSA. Besides, we show that from the quantification of the total mCSA, it is possible to affirm that the total muscular cross‐sectional area is larger in the groups of rodents submitted to LRT than in the control groups. Despite this, few skeletal muscles were quantified using this measure [FHL and plantaris]. In the studies in question, the total mCSA was estimated from calculations that took into account individual skeletal muscle mass, muscle fiber length, and muscle density (Hornberger & Farrar, 2004; Lee & Farrar, 2003). This outcome is capable of providing, at least indirectly, an adequate measure to estimate muscle hypertrophy in rodents. However, our analysis identified that not all muscle types show greater fCSA. This fact led us to indicate that, as is done in humans, the idea is to quantify both mCSA and fCSA. Also, few studies have investigated the effects of LRT on the type of muscle fibers alone (Deschenes et al., 2000, 2015). This situation can also converge to an interpretative error since it is possible to measure larger mCSA without a uniform change in the typology of muscle fibers (Bjørnsen et al., 2019).

The capacity for muscle hypertrophy depends fundamentally on the amount of mechanical stimulation imposed on skeletal muscles. Here, we demonstrate that there is great variability in the types of resistance training protocols being used by researchers. Interestingly, it seems that rats, like humans, are more susceptible to the volume of training (series × reps × load) than the level of effort imposed by the session (Lasevicius et al., 2019; Luciano et al., 2017; Lacerda et al., 2019; Tibana et al., 2017). Tibana et al. (2017) compared the effects of two different climbing volumes (4 vs. 8) on the mean gastrocnemius fCSA. Both groups trained with the same relative loads (50, 75, 90, and 100%), varying only the amount of climbs with each load. As expected, both groups demonstrated greater fCSA compared to the control group. However, the group with the highest volume (8 climbs) had the largest fCSA averages [Control = 1,800 ± 30; RT4 = 2,650 ± 60; RT8 = 3,050 ± 125]. Recent evidence has shown that LRT can stimulate muscle hypertrophy by increasing the phosphorylation of proteins such as mTOR (mammalian target of rapamycin), p70S6k (p70S6 kinase 1) and MyoD (myoblast determination protein 1) of the gastrocnemius (Ribeiro et al., 2017). Also, these same authors reported that in addition to the increase in cell signaling pathways for anabolism, there was also a reduction in the phosphorylation of proteins associated with muscle catabolism. Corroborating these data, Luciano et al. (2017) demonstrated that larger total loads (volume × intensity) are probably necessary to stimulate the greatest mean increases in fCSA, phosphorylation of mTOR, and their respective regulatory enzymes.

When analyzing the chronic response of each muscle, we demonstrated that the soleus, EDL, and plantaris do not seem to respond with the same magnitude of muscle hypertrophy as other muscles. One of the probable explanations for this fact can be directly associated with the particularities of each training protocol. Deschenes et al. (2015) failed to demonstrate a substantial increase in fCSA of soleus and plantaris muscles after seven weeks of LRT, 3x/week, using a protocol with 10 submaximal climbs. On the other hand, Krause Neto and Gama (2017) found higher averages of fCSA of the soleus and EDL muscles compared to the control group (5×/week, six climbs, 16 weeks). These divergences lead us to raise the hypothesis that some types of muscle may need higher training loads, while others need more significant volumes of training to hypertrophy. When analyzing these two cited studies, it is possible to verify that Deschenes et al. (2015) trained the rats for 21 sessions, while Krause Neto and Gama (2017) submitted their animals to 80 training sessions. Taking into account, it is plausible to suggest that some types of muscle may need more time to show higher hypertrophy levels than others. Recently, Padilha et al. (2019) demonstrated that the soleus muscle was responsive only to the protocol with the highest number of climbs (8–16 climbs/session).

On the other hand, muscles, such as plantaris and FHL, similarly hypertrophy in both types of training volume (high vs. moderate), showing a significant increase in protein synthesis. These data lead us to suggest that the results obtained here may have been influenced by the predominance of the type of muscle fiber in each muscle. However, due to the small number of studies that quantified the different types of muscle fibers, we were unable to investigate further each of these relationships. Despite this, Ribeiro et al. (2017) suggest that the lack of effect on soleus muscle hypertrophy could be directly linked to the inability of LRT to stimulate significant increases in muscle anabolism, even with a reduction in catabolism pathways. Also, weekly frequency, number of climbs, relative intensity, and duration of training are variables that can, in a certain way, affect the response of each type of muscle individually.

The mass of each isolated muscle is currently used as a marker of muscle hypertrophy in experimental models. However, our review results demonstrated that there is not necessarily a general relationship between the mass of individual muscles and the increase in CSA of muscle fibers. Despite this, the FHL muscle appears to demonstrate significant improvements in both fCSA and mCSA, in addition to its muscle mass (Hornberger & Farrar, 2004; Lee et al., 2016, 2018; Lee & Farrar, 2003). On the other hand, muscles like soleus seem to demonstrate a significant increase in their muscle mass, without necessarily affecting fCSA. This fact can, in part, be explained by a probable edematous muscle swelling induced by resistance training (Damas et al., 2016). Still, an inverse relationship can be seen regarding the TA and quadriceps femoris muscles, whose muscle mass is not different from those found in the control groups, but demonstrate more significant hypertrophic responses in their fCSA.

Finally, we demonstrated that MLCC increases regardless of the protocol used and the duration of the training. This interesting fact is easily explained since the animals have a high degree of sedentary lifestyle during the period of accommodation. Thus, by placing the animal under the physical stress of training, they obtain a rapid and significant functional gain. Corroborating, Deus et al. (2012) demonstrated that the LRT, without any additional load, is already sufficient to increase the load‐carrying capacity by the rodent, having its function enhanced by increasing the training load. In addition, morphological adjustments to peripheral nerves may explain, in part, the increase in muscle strength without necessarily increasing the size of the muscle or its fibers (Carbone et al., 2017).

## 5 CONCLUSION

The results obtained in this study led us to the following conclusions: (a) LRT is efficient in inducing hypertrophy of skeletal muscles, although this effect varies between different types of skeletal muscles, and; (b) the ability of rodents to carry load increases regardless of the nature and duration of the protocol used.

## CONFLICT OF INTEREST

All authors disclose any conflict of interest.

## AUTHOR CONTRIBUTIONS

The author's IL and WKN participated in the search for articles in the database, selection of articles, production, writing, and final reading of the manuscript. LA, VO, VG, GF, and EC participated in the search and selection of articles and final reading of the document. EG guided the entire process and the production of the final paper until submission.
